# Increase of Transmitted Drug Resistance among HIV-Infected Sub-Saharan Africans Residing in Spain in Contrast to the Native Population

**DOI:** 10.1371/journal.pone.0026757

**Published:** 2011-10-25

**Authors:** Gonzalo Yebra, Miguel de Mulder, María Jesús Pérez-Elías, José Antonio Pérez-Molina, Juan Carlos Galán, Jara Llenas-García, Santiago Moreno, África Holguín

**Affiliations:** 1 HIV-1 Molecular Epidemiology Laboratory, Microbiology Department, IRYCIS-Hospital Ramón y Cajal and CIBER-ESP, Madrid, Spain; 2 Infectious Diseases Department, Hospital Ramón y Cajal, Madrid, Spain; 3 Microbiology Department, Hospital Ramón y Cajal, Madrid, Spain; 4 HIV Unit, Hospital Doce de Octubre, Madrid, Spain; INSERM & Universite Pierre et Marie Curie, France

## Abstract

**Background:**

The prevalence of transmitted HIV drug resistance (TDR) is stabilizing or decreasing in developed countries. However, this trend is not specifically evaluated among immigrants from regions without well-implemented antiretroviral strategies.

**Methods:**

TDR trends during 1996–2010 were analyzed among naïve HIV-infected patients in Spain, considering their origin and other factors. TDR mutations were defined according to the World Health Organization list.

**Results:**

*Pol* sequence was available for 732 HIV-infected patients: 292 native Spanish, 226 sub-Saharan Africans (SSA), 114 Central-South Americans (CSA) and 100 from other regions. Global TDR prevalence was 9.7% (10.6% for Spanish, 8.4% for SSA and 7.9% for CSA). The highest prevalences were found for protease inhibitors (PI) in Spanish (3.1%), for non-nucleoside reverse transcriptase inhibitors (NNRTI) in SSA (6.5%) and for nucleoside reverse transcriptase inhibitors (NRTI) in both Spanish and SSA (6.5%). The global TDR rate decreased from 11.3% in 2004–2006 to 8.4% in 2007–2010. Characteristics related to a decreasing TDR trend in 2007-10 were Spanish and CSA origin, NRTI- and NNRTI-resistance, HIV-1 subtype B, male sex and infection through injection drug use. TDR remained stable for PI-resistance, in patients infected through sexual intercourse and in those carrying non-B variants. However, TDR increased among SSA and females. K103N was the predominant mutation in all groups and periods.

**Conclusion:**

TDR prevalence tended to decrease among HIV-infected native Spanish and Central-South Americans, but it increased up to 13% in sub-Saharan immigrants in 2007–2010. These results highlight the importance of a specific TDR surveillance among immigrants to prevent future therapeutic failures, especially when administering NNRTIs.

## Introduction

The presence of transmitted drug resistance mutations (TDR) in patients unexposed to highly active antiretroviral treatment (HAART) is a major problem in the management of HIV-1 infection. Several studies have described a high risk of virological failure to first therapy in patients harbouring resistance mutations conferring resistance to any of the drugs received. Nevertheless, first-treatment guided by initial resistance testing achieves similar efficacy in patients with primary drug resistance as in patients with wild-type virus [1]–[4]. Therefore, international guidelines recommend that initial treatment choice should depend on the HIV resistant test results prior to starting HAART [5]–[7].

Numerous works have analysed the TDR prevalence in Western Europe and the United States. After several years with a continuous increase in the TDR rate [8]–[10], the efficacy of HAART and the development of both new antiretroviral drugs and classes have led to stable [2], [11]–[14] or decreasing trends [15], [16] of TDR. However, the TDR trends could be intimately related to the antiretroviral programmes implemented in each region, but only few studies have reported TDR prevalences according to the origin of the patients [11], [17]. Given that immigrant patients account for a growing portion of the HIV-infected population in developed countries, the presence and trends of TDR among this subgroup should be explored in detail. In Spain, a third of newly HIV-diagnosed patients are immigrants [18]–[19], most of them coming from Central and South America or sub-Saharan Africa. The special socio-cultural characteristics of these populations as well as the antiretroviral policies established in their regions of origin may affect the transmission of resistant variants. Therefore, we analyze here the changes in the TDR rate through the last 15 years in a large set of HIV-infected naïve patients taking into account their origin.

## Materials and Methods

### Study population

A total of 732 HIV-1-infected patients diagnosed between 1996 and 2010 with at least one *pol* sequence prior to any antiretroviral treatment were included. Most (>98%) were under follow-up in different HIV/AIDS clinics in Madrid, Spain. The origin of the patients (i.e., self-reported place of birth) was: 292 native Spanish, 226 sub-Saharan Africans (SSA), 114 Central and South Americans (CSA), 26 East Europeans (including Russia), 20 West Europeans and North Americans, 3 North Africans, 2 Asians and 49 of unknown origin. The most frequent countries of origin for SSA were Equatorial Guinea (111 patients), Nigeria (28), Sierra Leone (10) and Liberia (7). For CSA, Ecuador (20), Argentina (15), Colombia (14), Brazil (13) and Cuba (13). For East Europeans Romania (10) and Russia (7). Finally, for West Europeans, the most frequent origins were France and Portugal (6 each). Both protease (PR) and reverse transcriptase (RT) sequences were available for 641 patients, only PR sequence for 89 patients and only RT for 2 patients. Most (495, 67.6% [CI: 64.2−71]) of the HIV-1 sequences included had been previously published [20]–[21]. The remaining were collected from Hospital Ramón y Cajal (n = 218) and Hospital Doce de Octubre (n = 19) in Madrid, Spain. This study was part of a project approved by the review board of the Hospital Ramón y Cajal Clinical Research Ethical Committee. It was designed to protect the rights of all subjects involved under the appropriate local regulations. To maintain subject confidentiality, a unique ID number was assigned to each specimen, and written consent obtained for each patient by clinicians.

### Drug resistance

The prevalence of transmitted drug resistance was defined according to the list of mutations for TDR surveillance as recommended by the World Health Organization [22] using the Calibrated Population Resistance tool [23]. Genotypic interpretation of these resistance mutations was evaluated using the Stanford HIVdb Algorithm [24], version 6.0.11. Resistance was normalized in three levels: susceptible (S), intermediate (I), and resistant (R).

### HIV-1 subtyping

HIV-1 subtypes and circulating recombinant forms (CRF) were identified by phylogenetic analysis of the *pol* sequences. The 2008 version of the subtype reference dataset provided by Los Alamos National Laboratory was used. At least two representative sequences of each 9 subtypes and the 43 CRF of HIV-1 group M available at the moment of the analysis were taken as references. DNA sequences were aligned using the ClustalX 2.0.11 program. The tree topology was obtained using the Neighbour-Joining method. The pairwise distance matrix was estimated using the Kimura two-parameter model within the DNAdist program, as implemented in the PHYLIP software package. Bootstrap re-sampling (1,000 data sets) of the multiple alignments was performed, with the bootstrap cut-off set at 700.

### Statistical analysis

Prevalences were expressed in percentage and 95% confidence interval (CI). Continuous variables were compared using the *t*-test. Categorical variables were compared using the chi-square test or Fisher's exact test if appropriate. Association between epidemiological, clinical and virological factors was analysed by univariate and multivariate logistic regression. The maximum model included the variables of origin of the patient, route of HIV infection, HIV subtype and time period of infection. Changes over time in the resistance prevalences were analysed using a chi-square test for trends (linear-by-linear association). Significance was set at p<0.05.

## Results

**Figure 1:**
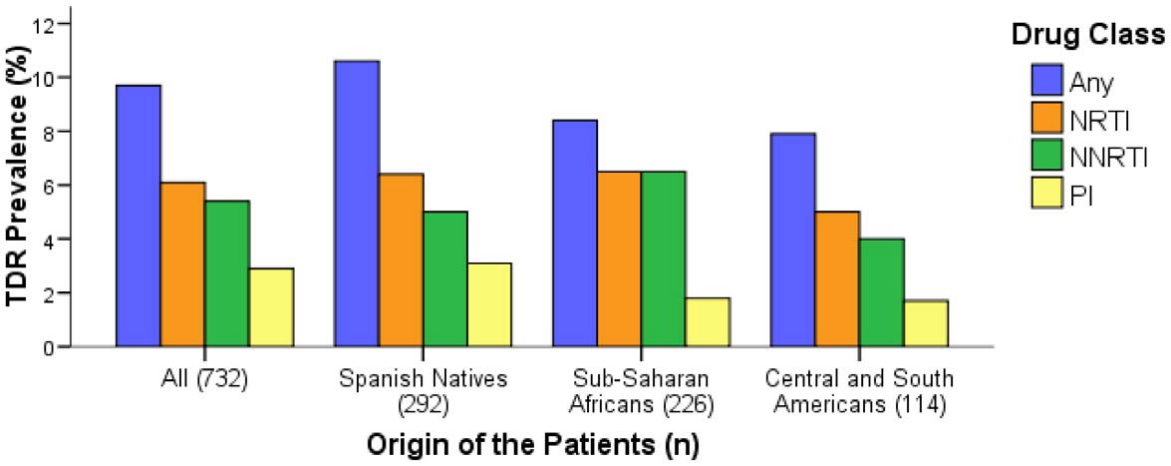
Prevalence of TDR to each drug class according to the origin of the patients. TDR, transmitted drug resistance; NRTI, nucleoside reverse transcriptase inhibitors; NNRTI, non-nucleoside reverse transcriptase inhibitors; PI, protease inhibitors; n, number of patients.

TDR prevalence (i.e., to any antiretroviral drug class) among the 732 patients diagnosed in Madrid, Spain, between 1996 and 2010 was 9.7% (CI: 7.6−11.8) (**Figure 1**). **Table 1** shows the main characteristics of the population comparing patients infected with wild-type viruses with those harbouring TDR. Regarding the antiretroviral drug class, TDR prevalence among patients in our study was 2.9% (CI: 1.7−4.1) for PR inhibitors (PI) (3.1% [CI: 1.1−5.1] in Spanish, 1.8% [CI: 0.1−3.5] in SSA and 1.7% [CI: −0.7−4.2] in CSA), 6.1% (CI: 4.2−7.9) for nucleoside reverse-transcriptase inhibitors (NRTI) (6.4% [CI: 3.6−9.3] in Spanish, 6.5% [CI: 2.8–10.2] in SSA and 5% [CI: 0.7−9.4] in CSA), and 5.4% (CI: 3.7−7.2) for non-nucleoside RT inhibitors (NNRTI) (5% [CI: 2.5−7.6] in Spanish, 6.5% [CI: 2.8–10.2] in SSA and 4% [CI: 0.2–7.9] in CSA). To assess the TDR prevalence according to the calendar year, sequences were grouped in three periods (2000-03, 2004-06 and 2007-10) according to the observed resistance trend by calendar year. Sequences obtained before year 2000 were included in the global estimates but excluded in the analyses due to their low number (n = 32) and representativeness since all were sequences from immigrant patients. In the whole study population, the TDR prevalence to any antiretroviral drug was 10.6% (CI: 5.3−15.9), 11.3% (CI: 7.8−14.8) and 8.4% (CI: 5−11.8) in those periods, respectively (*p* trend = 0.35). In the following paragraphs, the TDR prevalences and temporal trends in these periods are detailed according to different factors.

**Table 1:** Comparison of characteristics between patients infected with virus harbouring TDR and patients infected with wild-type virus.

	Total	With TDR	Wild-type	*p* value	OR (95% CI) in univariate analysis
Patients (n)	732	71 (9.7)	661 (90.3)	-	
Origin [n (%)]					
Spain	292 (39.9)	31 (43.7)	261 (39.5)	0.5	1
SSA	226 (30.9)	19 (26.8)	207 (31.3)	0.4	0.77 (0.42–1.40)
CSA	114 (15.6)	9 (12.7)	105 (15.9)	0.47	0.73 (0.33–1.58)
Other	100 (13.7)	12 (16.9)	88 (13.3)	0.38	1.15 (0.57–2.33)
Sex [n (%)]^a^					
Male	469 (68.6)	47 (69.1)	422 (68.5)	0.92	1
Female	215 (31.4)	21 (30.9)	194 (31.5)	0.92	1.67 (0.50–5.58)
Route of transmission [n (%)]^b^					
Heterosexual contact	269 (49.4)	23 (44.2)	246 (50.0)	0.43	1
Homo/bisexual contact	196 (36.0)	18 (34.6)	178 (36.2)	0.82	0.90 (0.46–1.78)
Injection drug use	68 (12.5)	9 (17.3)	59 (12.0)	0.27	1.58 (0.73–3.41)
Other	11 (2.0)	2 (3.8)	9 (1.8)	0.33	1.22 (0.70–2.12)
HIV-1 Subtype [n (%)]					
B	383 (52.3)	39 (54.9)	344 (52.0)	0.64	1
Non-B	349 (47.7)	32 (45.1)	317 (48.0)	0.64	0.89 (0.54–1.46)
Year of HIV-1 Infection [n (%)]					
<2000	32 (4.4)	0 (0)	32 (4.8)	-	0
2000-03	132 (18.0)	14 (19.7)	118 (17.8)	0.82	1
2004-06	318 (43.4)	36 (50.7)	282 (42.7)	0.19	1.08 (0.56–2.07)
2007-10	250 (34.1)	21 (29.6)	229 (34.6)	0.39	0.78 (0.38–1.58)

TDR, transmitted drug resistance; OR, odds ratio; CI, confidence interval; n, number of patients; SSA, sub-Saharan Africa; CSA, Central and South America.

a Data available for 684 patients.

b Data available for 544 patients.

c Data available for 541 patients.

### According to drug class

**Figure 2:**
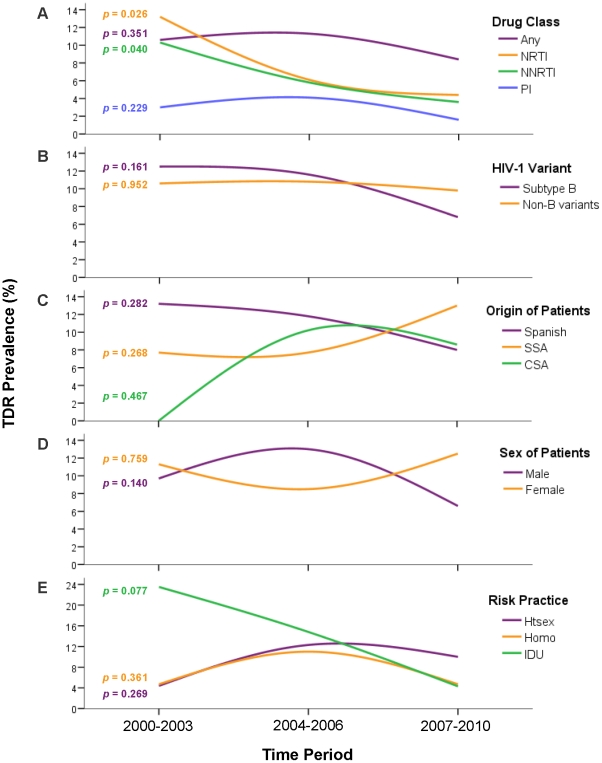
Temporal trends of TDR prevalence. The prevalences are detached according to A) drug class; B) HIV-1 variant; C) origin of the patients (as self-reported birth place); D) sex of the patients; and E) risk practice. TDR, transmitted drug resistance; NRTI, nucleoside reverse transcriptase inhibitors; NNRTI, non-nucleoside reverse transcriptase inhibitors; PI, protease inhibitors; SSA, sub-Saharan Africa; CSA, Central and South America; Htsex, heterosexual contact; Homo, homo/bisexual contact; IDU, injection drug use. The *p*-values shown next to the lines and sharing color code correspond to the chi-square test for trend in each case.

As observed in the global TDR prevalence, the presence of transmitted PI-resistance mutations reached a maximum in 2004-06 (4.1% [CI: 1.9−6.3]), and declined to its minimum value (1.6% [CI: 0−3.2]) in the last period (2007-10) (*p* trend = 0.23). On the other hand, rates of both NRTI- and NNRTI-resistance declined progressively and significantly along the three periods: 13.2% (CI: 5.2−21.3), 6.1% (CI: 3.4−8.7) and 4.4% (CI: 1.9−6.9), for NRTI (*p* trend = 0.03) and 10.3% (CI: 3.1−17.5), 5.8% (CI: 3.2−8.4) and 3.6% (CI: 1.3−5.9), for NNRTI (*p* trend = 0.04). This decline was more pronounced for NRTI-resistance mutations, getting closer to the NNRTI-resistance mutations rate in the last period (**Figure 2a**). Thus, transmitted resistance to RT inhibitors was two or threefold higher than that for PI in 2007-10 (*p* = 0.06).

### According to HIV-1 variant

Subtype B was found in 383 (52.3%, [CI: 48.7–55.9]) patients, meanwhile non-B subtypes and recombinants infected 349 (47.7% [CI: 44.1–51.3]) patients. Among them, the most frequent variants were: CRF02_AG (128 patients), subtype A (36), subtype G (35), CRF12_BF (23) and subtype C (22). Although global TDR prevalence was similar in subtype B and in non-B variants (10.2% [CI: 7.1–13.2] *vs.* 9.2% [CI: 6.1–12.2], *p* = 0.64) their TDR temporal trend was different (**Figure 2b**): in subtype B, TDR declined from 12.5% (CI: 3.1−21.9) in 2000-03 to 6.8% (CI: 2.3–11.4) in 2007-10 (*p* trend = 0.16), but in non-B variants this prevalence remained stable (9.5% [CI: 3.2–15.8] and 9.8% [CI: 4.7–14.8], respectively [*p* trend = 0.99]). Of note, TDR was higher in non-B than in subtype B (9.8% [CI: 4.7–14.8] *vs*. 6.8% [CI: 2.3–11.4], *p* = 0.54) in the last period.

### According to the origin of patients

Global TDR prevalences were 10.6% (CI: 7.1–14.1) in native Spanish, 8.4% (CI: 4.8–12) in SSA and 7.9% (CI: 2.9–12.8) in CSA (**Figure 1**). Regarding the temporal trends in each population (**Figure 2c**), TDR declined constantly in Spanish natives: 13.2% (CI: 2.41–23.9) in 2000-03, 11.8% (6.7–17) in 2004-06 and 8% (CI: 2.7–13.3) in 2007-10 (*p* trend = 0.28). In CSA, no TDR mutations were detected in 2000-03, but TDR reached similar rates as in natives in the following periods: 10.2% (CI: 2.5–17.9) in 2004-06 and 8.6% (CI: −0.7–17.8) in 2007-10 (*p* trend = 0.47). In contrast, among SSA the TDR prevalence remained stable in 2000-03 and 2004-06 (7.7% [CI: 1.2–14.2] in both cases) and rose up to 13% (CI: 5.1–21) in 2007-10 (*p* trend = 0.27). Thus SSA presented the highest TDR frequency in the last period. Finally, TDR prevalence was very high among East Europeans (5/26, 19.2% [CI: 4.1–34.4]) and West Europeans (3/20, 15% [CI: −0.6–30.6]), although the low number of patients did not allow to differentiate the prevalence according to temporal trend.

### According to sex of the patients

Most (68.6% [CI: 65.1–72]) of the subjects with available data were males (**Table 1**). Global TDR prevalence was similar between them and females (10% [CI: 7.3–12.7] *vs.* 9.8% [CI: 5.8–13.7], respectively, p = 0.97). Although this similarity was observed in 2000-03 (9.7% [CI: 2.3–17] *vs.* 11.3% [CI: 3.4–19.2]), TDR increased among male patients but decreased among females in 2004-06 (13% [CI: 8.7–17.4] *vs.* 8.5% [CI: 1.4–15.6], *p* = 0.46) and vice versa in 2007-10 (6.6% [CI: 2.8–10.3] *vs.* 12.5% [CI: 4.9–20.1], *p* = 0.21).

### According to route of HIV infection of the patients

Half (49.4% [CI: 45.2–53.6]) of the patients with known route of transmission had been infected through heterosexual intercourse (**Table 1**). TDR prevalence was higher (13.2% [CI: 5.2–21.3]) in injection drug users (IDU) and similar in those infected through homo/bisexual (9.2% [CI: 5.1–13.2]) and heterosexual (8.5% [CI: 5.2–11.9]) intercourse. Regarding the temporal trend, there was a drastic decrease in the TDR rate among IDU (n = 68), the most common risk practice in Spain until the late 90 s. This rate was 23.5% (CI: 3.4–43.7; 4/17 patients) in 2000-03, 14.8% (CI: 1.4–28.2; 4/27) in 2004-06 and 4.3% (CI: −4–12.7; 1/23) in 2007-10 (*p* trend = 0.08). Among homo/bisexuals, the prevalence rose from 2000-03 (4.7% [CI: −4.3–13.9]) to 2004-06 (12.6% [CI: 6.44–18.8]) but decreased to the initial level in the last period (4.7% [CI: −0.5–10]) (*p* trend = 0.36). Finally, the TDR rate in heterosexuals seemed to stabilize after an increase from 2000-03 (4.4% [CI: −0.5–9.3]) to 2004-06 (12.3% [CI: 4.8–19.9]), and was the risk practice with the highest TDR prevalence in 2007-10 (10% [CI: 4.1–15.9]) (*p* trend = 0.32).

### Patients infected with viruses harbouring TDR mutations

**Figure 3:**
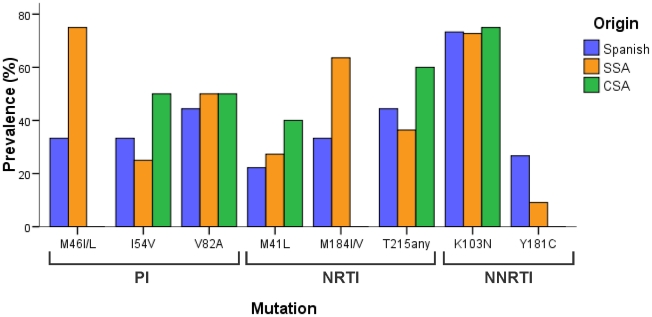
Prevalence of individual mutations among the patients infected with HIV-1 variants harbouring any TDR. SSA, sub-Saharan Africa; CSA, Central and South America; PI, protease inhibitors; NRTI, nucleoside reverse transcriptase inhibitors; NNRTI, non-nucleoside reverse transcriptase inhibitors.

Among the 71 patients harbouring TDR mutations (**Table 1**), 31 were Spanish, 19 SSA, 9 CSA, 5 East Europeans, 3 West Europeans, 1 North African and 3 of unknown origin. In three quarters (53, 74.6% [CI: 64.2–84.8]) of the cases, TDR affected only a single drug class. However, in 6 patients (4 Spanish, 1 SSA and 1 CSA) a triple-class resistance was found. The pattern of TDR mutations was different for NRTI- and PI-resistance according to the origin (**Figure 3**). Among SSA, RT-M184I/V and PR-M46L were the most frequent mutations, respectively, meanwhile in both Spanish natives and CSA, RT-T215rev and PR-V82A were the most prevalent substitutions. For NNRTI-resistance, K103N was the most frequent mutation in all groups, although its prevalence was higher in SSA (4.7% [CI: 1.5–7.8]). In fact, K103N was the most prevalent mutation in all periods, and it was the mutation found in a third of the cases (15/45) where a single TDR mutation was found. The temporal trend of specific mutation prevalences showed significant reductions from 2000-03 to 2007-10 for tymidine-analogue mutations together (8.8% [CI: 2.1–15.6] to 2.4% [CI: 0.5–4.3], *p* trend = 0.03) and K103N (10.3% [CI: 3.1–17.5] to 3.2% [CI: 1–5.4], *p* trend = 0.02) and non-significant reduction for M184I/V (4.4% [CI: −0.5–9.3] to 2.4% [CI: 0.5–4.3], *p* trend = 0.67). In the logistic model (**Table 1**), there was no significant difference in the prevalence of TDR according to sex, year or route of HIV-1 infection or origin of the patients.

### Clinical implications of TDR

**Figure 4:**
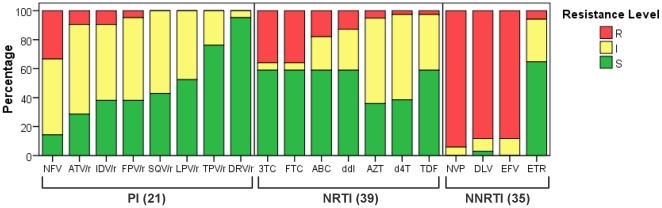
Predicted susceptibility to antiretroviral drugs of the 71 viruses carrying TDR mutations. Susceptibility was estimated according to the HIVdb Interpretation Algorithm (Stanford University, Palo Alto, CA, USA). TDR, transmitted drug resistance; PI, protease inhibitors: nelfinavir (NFV); atazanavir/r (ATV/r), indinavir/r (IDV/r), fosamprenavir/r (FPV/r), saquinavir/r (SQV/r), lopinavir/r (LPV/r), tipranavir/r (TPV/r) and darunavir/r (DRV/r), where “/r” indicates co-administration with low-dose ritonavir (RTV) for pharmacological “boosting”. NRTI, nucleoside reverse transcriptase inhibitors: lamivudine (3TC), emtricitabine (FTC), abacavir (ABC), didanosine (ddI), zidovudine (AZT), stavudine (d4T), tenofovir (TDF). NNRTI, non-nucleoside reverse transcriptase inhibitors: nevirapine (NVP), delavirdine (DLV), efavirenz (EFV), etravirine (ETR); R, high level resistance; I, intermediate resistant; S, susceptible.

The analysis of the genotypic resistance interpretation of the 71 *pol* sequences harbouring TDR (**Figure 4**) revealed that especially in the case of PI there are options to select a fully active drug even in patients carrying PI-resistance mutations if the treatment choice is guided by the resistance test. Actually, none of the 21 patients with PI-resistance mutations presented high resistance levels to all PI tested due to their susceptibility to new-generation drugs like darunavir or tipranavir. Among the 39 patients with NRTI-resistance mutations, only 5 (12.8% [CI: 2.3–23.3]) presented some level of resistance to all NRTI included. Furthermore, 14 (35.9% [CI: 20.8–50.9]) and 16 (41% [CI: 25.6–56.5]) were fully susceptible to the most used NRTI combinations, emtricitabine/tenofovir and lamivudine/abacavir. Regarding NNRTI-resistance, none of the 34 patients with resistance mutations was fully susceptible to efavirenz and nevirapine, the most used drugs of this class. Only etravirine would have some level of activity in these patients.

### Differences in HIV viral load

Patients infected with HIV variants harbouring TDR presented a significantly lower mean viral load compared to those infected with wild-type HIV (4.1 log [SD: 0.83] *vs.* 4.4 log [SD: 0.83] RNA-HIV-1 copies/ml, *p* = 0.03). This result was also obtained when comparing patients infected with viruses carrying or not the mutation M184I/V (3.9 log [SD: 0.86] *vs.* 4.4 log [SD: 0.83], *p* = 0.04). In addition, according to sex, male patients presented a significantly higher viral load than female patients (4.5 log [SD: 0.79] *vs*. 4.2 log [SD: 0.89], p<0.001) at diagnosis time. There were no differences in plasma HIV viraemia according to origin, risk practice, HIV infection year or HIV subtype in the study population.

### Transmission clusters

Phylogenetic analyses only revealed a cluster of drug-resistance mutations transmission. This involved a native Spanish and a Chilean diagnosed in 2006 in the same clinic and infected with three-drug class resistant subtype B viruses. Both specimens harboured mutations F53L and L90M at PR and M41L, K103N, L210W and T215D at RT.

## Discussion

This study explores the evolution of TDR rates through 15 years (1996–2010) in a large set of HIV-infected naïve patients diagnosed in Spain taking into account different factors: drug class, viral variant, origin of the patient, route of infection and sex. The global TDR prevalence reported in this work (9.7%) is in agreement with the most representative European studies, where this rate has stabilized around 10% [1], [11], [12], [15]. In the USA, the TDR prevalence has traditionally been higher, but it also has started to stabilize [4], [25]. According to our results, the rate of transmitted NRTI-resistance is clearly dropping in Spain until reaching the levels of NNRTI-resistance, as already reported in other developed countries [11], [15]. In addition, in three quarters of the cases the transmission of resistance is limited to a single class.

The stabilization of TDR rates is partially due to the high efficacy of the HAART in Europe, where there is universal access to this treatment. However, in other regions where treatment is not available to all HIV-infected people on a regular basis, emergence and transmission of HIV resistant variants is likely to happen. Thus, it could be assumed that the TDR trend previously reported in developed countries will be observed in developing regions as treatment programmes are implemented. According to this, low TDR prevalences found in the last years in regions with shorter HAART tradition [26]–[27] are expected to be followed by a peak as seen in Europe around years 2002-03. This has been reported in African regions with an older treatment scale-up [28]–[30]. Here, we describe a growing TDR prevalence among sub-Saharan patients (SSA) in contrast to the native HIV-infected population and the general trends reported in West Europe. A high rate of losses to follow-up has been described among SSA under treatment in Spain probably due to a low educational level and their high geographic mobility looking for work [31]. Due to an ineffective follow-up, drug-resistance variants can be emerging and circulating among treated SSA and eventually producing TDR events within this subgroup. Given that SSA patients occasionally come to Spain to receive antiretroviral treatment and return afterwards to Africa, transmission of HIV resistant variants within this collective could be happening either in Africa or in Spain. Thus, the observed increasing TDR trend among infected SSA in our study population, the high prevalence of HIV-1 non-B variants in that collective, and the different NRTI-resistance TDR pattern found in SSA than in native Spanish, would strongly suggest that SSA in Spain could have acquired the infection in their countries of origin or in Spain through infected Africans carrying resistant viruses.

On the other hand, we observed an absence of TDR in 2000-03 among the 18 CSA in our cohort. However, TDR rates reached similar levels as in native Spanish in the following periods. This could be due to the low number of CSA patients included in the first period or to the limited scale up access to treatment in these regions. Nowadays, TDR trends in CSA patients are expected to be similar to trends among Spanish because antiretroviral therapy programmes have been working in most of these countries for at least a decade [32]. Also the presence of non-B variants typical from certain regions of Central and South America (CRF12_BF in Argentina and Chile and CRF23_BG in Cuba) among CSA patients harbouring resistant strains suggests that these infections probably occurred before arriving to Spain. Despite their low representation, both East and West Europeans (26 and 20 patients, respectively) showed TDR prevalences beyond 15%. Nevertheless, these patients infected with resistance variants were diagnosed before 2006, and were IDU in most cases, both characteristics related to a higher TDR prevalence according to our results.

Focusing on the HIV subtype, we describe a higher TDR prevalence in the last study period (2007-10) in patients carrying non-B variants *versus* subtype B. This was due to the TDR decrease among subtype B in contrast to its stabilization among non-B variants. This is in agreement with other studies reporting growing TDR rates in non-B *versus* subtype B [20], [33]. However, most studies [11]–[13], [15] report higher TDR rates in subtype B, although all of them describe the absolute prevalences in the entire study period and not the temporal trends according to the subtype. Our results could be misleading due to the high prevalence of non-B subtypes and recombinants among SSA patients. Actually, excluding SSA patients, non-B variants showed a decreasing TDR prevalence from 15.8% to 6.1% in the first and last period, respectively (results not shown). Therefore the increase was probably due to the origin, not to the subtype. On the other hand, the correlation between HIV subtype and birth place of the patients could also have confused the different mutational pattern described: the preferential presence of NRTI-resistance mutation M184I/V in SSA compared to T215revertants in Spanish and CSA could appear to be associated to the infection by non-B variants and subtype B, respectively, as reported in other countries [34], [35]. However, the different antiretroviral strategies implemented in these regions could also be a probable cause of this different mutational pattern in the studied naive HIV-1-infected patients carrying resistant viruses.

Our observation of a lower viral load among patients infected with variants carrying M184I/V has previously been described [36] due to the fitness reduction related to its acquisition. In addition, the lower viral load in female *versus* male at baseline has already been observed [37]. Regarding the analysis according to the route of infection, the decrease of the TDR rate among IDU across the years is a consequence of the evolution of HIV epidemics in Spain and a better treatment compliance among the IDU population. In the late 90 s the use of injecting drugs was by far the first route of HIV infection, but clearly decreased while HIV transmission due to unprotected sexual contacts increased [19].

According to the genotypic resistance interpretation, the TDR mutations found in 71 patients mainly affect the NNRTI-based therapy, the first choice in Spain for naïve patients [7], due to the presence of K103N, the most frequent mutation in the study especially among SSA. Despite its transmission as a singleton mutation, K103N seriously compromises the use of efavirenz or nevirapine. Only etravirine, the second-generation NNRTI approved solely for salvage regimens, could have some level of activity in these patients. Therefore, resistance testing prior to first-line treatment choice is strongly recommended. In developed countries there are therapeutic options as new generation NNRTI, PI or even new classes as integrase or entry inhibitors. Unfortunately, all these options are not available in developing regions where NNRTI-resistance among naïve patients is rising [28]. Interestingly, the highest NNRTI-resistance prevalence in our study was found among SSA, although in some cases this could possibly be explained by unrecorded, prophylactic use of nevirapine in Africa. Our results also suggest that PI could be active in most naïve patients carrying PI-resistance mutations if the treatment choice were guided by the resistance test.

The main limitation of this study is the restricted number of patients which prevents a solid statistical support of the TDR trends, for instance analyzing East and West Europeans living in Spain. The delimitation of the study periods could also be a source of bias despite the fact they were selected to reflect the trend by calendar year as accurately as possible. In addition, sub-Saharan patients might be overrepresented due to the special care they receive in the Tropical Medicine Units ascribed to the clinics included in the work, especially patients from Equatorial Guinea, a former Spanish colony located between Cameroon and Gabon. Nevertheless, since the year 2000 the immigration in Spain has increased exponentially, particularly in Madrid, where 17% of the general population were foreigners in 2010. Finally, the surprisingly high rate in native Spanish of RT-M184I/V (4.4%), a mutation rapidly cleared due to its cost in terms of viral fitness [38], might reflect an unrecorded previous treatment exposure in some patients.

In summary, TDR prevalence in Spain follows the general trend in Western Europe. The TDR rate is decreasing especially for NRTI-resistance. However, the TDR trends during 2000–2010 differed according to the origin of the patients. Whereas in native Spanish and Central-South Americans the TDR rate is decreasing, we report for the first time an increasing TDR prevalence among sub-Saharan patients diagnosed in Spain, who form the collective with the highest TDR prevalence in Spain since 2007. This could be due to the special socio-cultural characteristics of these patients, which may compromise antiretroviral treatment compliance and follow-up. Since TDR is expected to increase in developing regions as treatment is implemented, the presented results highlight the importance of a specific TDR surveillance among immigrants living in high-income countries to prevent future therapeutic failures, especially when administering NNRTI due to the high K103N prevalence.
